# “It’s legal, now what?” development, implementation, and evaluation of interdisciplinary cannabis education for healthcare trainees

**DOI:** 10.1186/s42238-025-00321-8

**Published:** 2025-09-26

**Authors:** Sean P. Cronin, Josue Cruz, Elena Cameron, Sabrina Azemar, Steven Dudley, Tally M. Largent-Milnes, Benjamin R. Brady, Jessica S. Wallace, Margie R. Arnett, Stephen M. Dahmer, Mohab M. Ibrahim, Alyssa R. Padilla, Todd W. Vanderah, Jennifer S. De La Rosa

**Affiliations:** 1 Comprehensive Center for Pain & Addiction, University of Arizona, Tucson, AZ USA; 2 Mel and Enid Zuckerman College of Public Health, University of Arizona, Tucson, USA; 3 Arizona Poison and Drug Information Center, University of Arizona, Tucson, USA; 4 R. Ken Coit College of Pharmacy, University of Arizona, Tucson, USA; 5 Department of Pharmacology, College of Medicine – , University of Arizona, Tucson, USA; 6 College of Health and Human Services, Western Michigan University, Kalamazoo, USA; 7 Center for Transformative Interprofessional Healthcare, University of Arizona, Tucson, USA; 8 Andrew Weil Center for Integrative Medicine, University of Arizona, Tucson, USA; 9 Department of Family and Community Medicine, College of Medicine – Tucson, University of Arizona, Tucson, USA; 10 Department of Anesthesiology, College of Medicine – Tucson, University of Arizona, Tucson, USA

**Keywords:** Cannabis, Interprofessional, Curriculum, Training, Continuing Education, Implementation, Evaluation, Healthcare Professionals, Person-Centered, Patient-Provider Communication

## Abstract

**Background:**

Healthcare professionals are motivated to improve their cannabis knowledge—yet few training opportunities exist. The unique legal status of cannabis, lack of rigorous research, and rapid expansion of consumer demand present challenges to the development and implementation of cannabis education for healthcare professionals and trainees. As a result, an alarming gap in quality of care is developing: health care professionals across disciplines lack the knowledge needed to counsel their patients, even as cannabis use rapidly accelerates.

**Methods:**

We aimed to address the gap by developing and implementing an interprofessional cannabis training for healthcare trainees. Considering the challenges to development and implementation of cannabis training, we identified 4 implementation strategies to maximize training quality, uptake, and utility*:* 1) incorporating a diverse array of scientific expertise and perspectives in curriculum development; 2) offering a comprehensive, evidence-based treatment of potential risks and potential benefits; 3) using an interprofessional training format; 4) adopting a person-centered lens with special emphasis on patient-provider communication. A post-survey evaluated implementation success and intermediate outcomes in trainee attitudes and behavioral intentions that would suggest high potential to deliver healthcare improvements at scale.

**Results:**

The virtual training *“It's Legal, Now What? Cannabis Epidemiology, Treatment, and Safety Recommendations”* was successfully implemented; since 2023 a total of 345 trainees in Pharmacy, Nursing, Public Health, and Medicine have earned certificates. Evaluation results are encouraging: 90% agree the training addressed a training need in their current role, 83% agree it should be required for trainees in their profession, 98% and 96% agree it comprehensively addressed potential risks and benefits, respectively, 94% agree it was inclusive of diverse perspectives, 94% agree it improved their knowledge of community resources, 96% report improved ability to respond to patients interested in cannabis, 96% report greater likelihood of providing information on cannabis to others, and 97% of trainees agree they learned information that would help them in their work or community.

**Conclusions:**

We present our training development process, implementation strategy, and evaluation as an adaptable model for contexts where both recreational and medical use of cannabis are legal.

**Supplementary Information:**

The online version contains supplementary material available at 10.1186/s42238-025-00321-8.

## Background

Cannabis use is rapidly accelerating globally; (Alevizopoulos et al., 2024) in the United States, cannabis has recently outpaced alcohol in the prevalence of recreational daily use. (Caulkins n.d.) At the same time, cannabis continues to occupy contested and frequently stigmatized terrain across legal, (Alharbi 2020, Ebling et al., 2025, Hossain & Chae 2024) scientific, healthcare, and public health domains. Its unique social position likely presents barriers to the effective development and implementation of health professional training on cannabis. 

At present, few cannabis training opportunities exist. (Zolotov et al., 2021) The available evidence suggests that fewer than half of providers may ever have received any formal training on medical or recreational use of cannabis. (Zolotov et al., 2021, Calcaterra et al., 2020) As a result, health trainees and professionals report a persistent information gap that leaves them unprepared to counsel patients on even the most basic cannabis-related questions. (Calcaterra et al., 2020) Furthermore, few providers initiate conversations with patients about cannabis use, (King et al., 2024) and, perhaps due to anticipated stigma, few patients who use cannabis volunteer this information to their providers. (Lapham et al., 2022).

Across disciplines, health professionals’ desire to improve their cannabis knowledge and competence to better meet patient needs has been well-documented. At the same time, given the lack of rigorous research, (Sznitman & Zolotov 2015) healthcare providers may struggle with holding competing lenses of risk and benefit, and disentangling the conditions under which cannabis is helpful or not helpful. (Zolotov et al., 2019).

In particular, the literature suggests that training is needed on topics such as therapeutic dosing, drug-drug interaction, legal concerns around access, adverse effects, and how to engage in meaningful conversations with patients and colleagues (Kruger et al., 2022, Baral et al., 2024, Ware & Ziemianski 2015) We developed and implemented an interprofessional health care training on cannabis. We present our implementation strategy and preliminary outcomes as an adaptable model suitable for any context where recreational and/or medical use of cannabis is legal.

The lack of cannabis training for health professionals constitutes a concerning gap in quality of care. Without action, patients will continue being left to fend for themselves amid public health messaging that is largely led by industry (Salter et al., 2022, Boehnke et al., 2024)– instead of scientists, clinicians, and public health professionals citing rigorous research.

## Methods

### Implementation strategies

We identified four implementation strategies to mitigate foreseeable challenges to implementation of cannabis training.

#### Incorporating diverse scientific and clinical cannabis expertise in project development

Medical and recreational use of cannabis are regularly stigmatized, (King et al., 2024, Dahlke et al., 2024, Reid 2020, Borojevic & Söhner 2025) as is cannabis research and the dedicated researchers who pursue it. (Sohn 2015, Martin-Willett et al., 2023) The burden of proof may be higher in cannabis research than most other topics, due to the belief that cannabis research is at an elevated likelihood to be biased, (Sohn 2015, National Academies of Sciences 2017, Grundy et al., 2023, Purcell et al., 2022, Hutchison et al., 2019) instead of scientifically objective and evidence-based. (Wheeldon & Heidt 2023) We committed to avoiding any appearance of bias by assembling a wide range of experts looking at cannabis from multiple perspectives.

#### Comprehensive and evidence-based treatment of cannabis risks and benefits

Health messaging around cannabis has historically been ideologically polarized, one message emphasizes cannabis safety and therapeutic potential, and the other invoking its risks. Source material for the curriculum included primary research, peer-reviewed literature, state-level and locally responsive data from Poison Control, as well as materials from the Center for Disease Control (CDC). Materials were used to develop a comprehensive, robust, and evidence-based treatment of potential risks and potential benefits as they related to cannabis epidemiology, clinical considerations, and person-centered care. We have provided a list of sources used in content development (Supplemental Table 1).

#### Interprofessional trainee recruitment and training format

Evidence shows that Healthcare professionals’ attitudes toward cannabis vary widely by setting and specialty. (Rønne et al., 2021) As a substance simultaneously approved for medicinal and recreational use, cannabis exists at the intersection of many health disciplines. Therefore, addressing cannabis training needs would logically benefit from a interprofessional training approach. (Earnest 2018) To reach a target audience of healthcare trainees from various disciplines, we partnered with the Center for Transformative Interprofessional Healthcare (CTIPH), which offers interprofessional education event operational infrastructure and support in distributing event information to healthcare trainees across the University of Arizona Health Sciences. Recruitment of students consisted of emails sent to a listserv of healthcare trainees at three universities highlighting a menu of several interprofessional training opportunities on a range of topics. Trainees self-select their preferred events. Depending on their college, students are required to complete either two or four interprofessional training events over the course of their degrees. The operational partnership with CTIPH made it possible to provide the training free of charge.

#### Special emphasis on person-centered communication

Patients use cannabis for a wide variety of reasons, both recreational and therapeutic. Therefore, public messaging and patient conversations on the topic of cannabis can benefit from a person-centered approach.(Lin et al., 2024, Bosley et al., 2023, Livingston et al., 2025, Kabakov et al., 2022) The training highlighted patient-provider communication which emphasized autonomy, goals, and making active choices rather than admonishments or one-size-fits-all recommendations.

### Curriculum design

Collaborating partners included Arizona Poison and Drug Information Center (AZPDIC), which operates of a 24-h hotline answering questions about cannabis exposure and drug interactions and leads an active cannabis education initiative providing information designed for use by the general public. The committee met monthly for four months to develop a two-hour interprofessional training. Specific learning objectives were developed in accordance with the Interprofessional Education Collaborative's (IPEC) report for core competencies as well as the WHO Framework for Action on Interprofessional and Collaborative Practice.(Gilbert et al., 2010).

### Evaluation design

We used a retrospective post-survey evaluation design. This approach is feasible even amid resource and operational constraints, minimizes respondent burden, and is useful in mitigation of response-shift bias at baseline that is associated with pre- post- designs in evaluation of training. (Howard 1980).

We were mindful of the possibility that students who were most interested in receiving cannabis training would be most likely to rate the training positively. All University of Arizona healthcare professions trainees are required to participate in at least two Interprofessional Education Events during the course of their degree program. Since the cannabis course was newly offered in Year 1, we reasoned that selection bias would be most prominent in year 1, attracting those students were who were most enthusiastic about the initial offering. The consistency of mean Likert values comparing Years 1 and 2 was examined (independent samples t-test).

Survey items measured the quality of training implementation as experienced by trainees, and their behavioral intentions and attitudes following the survey. Post-surveys included 5-point Likert scale items ranging from “Strongly Agree” to “Strongly Disagree”. All Likert items/responses are descriptively presented in Table 2. Post-surveys also included free text boxes for qualitative comment, for example “Name 3 things you learned in this IPE”. (Qualitative items are listed in Table 3). Qualitative responses were thematically summarized alongside the corresponding Likert items.

### Data collection

Data were collected within one week of the training using a retrospective post-survey collected in Qualtrics, a HIPPA-compliant data collection and survey tool used to administer and store data from post-surveys. Descriptive evaluation results are presented below.

## Results

### Training curriculum

The finalized cannabis training curriculum consisted of 4 structured units: 1) *public health and epidemiology*, 2) *clinical considerations*, 3) *person-centered communication,* and 4) *a team-based scenario*.

#### Public health and epidemiology

This unit examined cannabis trends using current local and national epidemiological information. Topics included: prevalence and reasons for use, distribution of use across all ages, increasing potency of marketed products. Prevalence of adverse effects were shown using data from poison control calls which include data collected on type and source of product ingested. Intended takeaways for patient safety include the trend toward higher potency products, and the importance of reliable product testing and accurate labeling to avoid contaminants and unwanted toxicological effects, and purchase from licensed dispensaries rather than the online or street market which may carry higher risks. (Commissioner 2024, de Oliveira et al., 2023, Lozier et al., 2019, Kelkar et al., 2018).

#### Clinical considerations

Healthcare providers will increasingly need to consider cannabis use by patients as part of clinical decision-making. This unit presented up-to-date scientific information regarding drug-drug interactions, FDA-approved medications fabricated with synthetic or cannabis-derived cannabinoids, and the respective indications and contraindications for both THC and CBD. It examined current qualifying conditions for state-issued medical cards, risk factors (age, pregnancy status, comorbidities (i.e. substance use disorders) and polypharmacy), and the value of including cannabis use in patient screenings and in the electronic health record (EHR). Claims commonly made by the cannabis industry that are not supported by the current scientific evidence were clearly highlighted.

#### Person-centered clinical communication

Communication skills taught in this unit included engaging with patients in a manner that reduces stigma, offers balanced information, and centers collaboration with the patient to maximize their health outcomes. Person-centered techniques for behavioral change such as motivational interviewing were introduced and modeled during a Stoplight conversational activity. The activity modeled collaboration with patients by having patients identify their green, yellow and red lights for their personal wellness goals relating to cannabis.

#### Interprofessional team-based scenario

The interactive team-based scenario allowed trainees a chance to apply knowledge gained during the didactic portion. Students were strategically assigned to break-out groups of 4–6 students with at least 2 health disciplines represented in each group. Although discussion remained student-led, each group was overseen by a member of faculty or staff from the colleges of medicine, nursing, pharmacy, or public health to provide additional context, offer clinical insights, and redirect as needed. To encourage interprofessional communication, the scenario text and guiding questions were designed to activate the unique insights pertinent to the variety of health disciplines in attendance. One scenario consisted of an older individual presenting at the Emergency Department with symptoms mimicking overstimulation of the parasympathetic nervous system (consistent with responses to pesticide contamination (Dryburgh et al., 2018, Montoya et al., 2020, Jameson et al., 2022) and who did not mention cannabis when answering questions on current medications. Guiding questions inserted at key moments in the scenario prompted student reflection on the patient’s source of cannabis, possible etiologies for the medical distress including pesticide contamination or drug-drug interactions, how stigma could interfere with patient care, and how health professionals could best counsel the patient after symptoms were stabilized. Students returned to the main session and shared personal reflections on how it felt to represent their discipline and hear from other disciplines during this interprofessional experience.

### Implementation and behavioral intentions

The training was delivered twice in November for two consecutive academic years, for a total of 4 sessions. Each 2-h training was offered using a virtual (Zoom) format. To date, 358 students have taken part in the Cannabis training; 345 students completed the evaluation surveys, resulting in a 96% response rate.

#### A wide range of expertise was successfully incorporated in project development

To design the cannabis training, a curriculum development committee was formed consisting of an interprofessional team of cannabis researchers from pharmacology, toxicology, family and community medicine, integrative medicine, pharmacy, addiction medicine, public health professionals, and health educators.

#### Interprofessional trainees participated and the training was experienced as relevant to professions

**Fig. 1:**
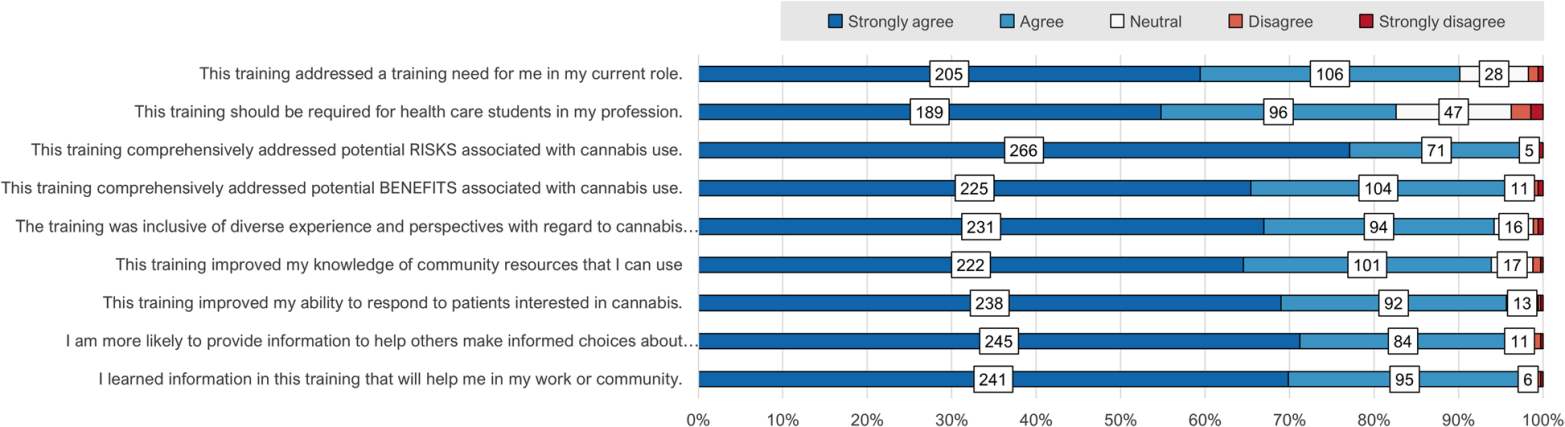
Evaluation of implementation and impact of an interprofessional cannabis training, University of Arizona

Students represented various health science pre-professionals, including pharmacists, nurses, public health educators, medical doctors, and individuals pursuing careers in athletic training and nutritional sciences (Table 1). Most students (90.1%) endorsed “Agree” or “Strongly agree” that “*This training addressed training need for me in my current role*” (Fig. 1**, **Table 2). 82.6% of students selected “Agree” or “Strongly Agree” with the statement that “*This training should be required for healthcare students in my profession*” **(**Fig. 1**, **Table 2). Qualitative responses note the benefit of aligned messaging and knowledge across disciplines, particularly between team-based patient interactions in clinical settings and public health promotion in community settings (Table 3).

**Table 1:** Participant characteristics of an interprofessional cannabis training, University of Arizona

	***n***	**%**
**Age**		
*18–24*	204	59%
*25–34*	109	32%
*35–44*	24	7%
*45–54*	8	2%
**Sex/Gender**		
*Male*	84	24.5%
*Female*	256	74.6%
*Another Sex/Gender*	2	0.6%
*Did not answer*	1	0.3%
**Race/Ethnicity**		
NH* American Indian or Alaska Native	4	1.1%
*NH Asian*	37	10.6%
*NH Black or African American*	22	6.3%
*Hispanic or Latinx/o/a or Chicano/a*	80	22.9%
*NH Middle Eastern/North African*	10	2.9%
*NH Native Hawaiian or Other Pacific Islander*	2	0.6%
*NH White*	147	42.1%
*Multiracial or two or more races*	36	10.3%
*Did not answer*	11	3.2%
**Discipline**		
*Pharmacy*	118	34.2%
*Nursing*	169	49.0%
*Public Health*	30	8.7%
*Medical Doctor*	5	1.4%
*Another discipline*	23	6.7%
**Student year in current degree**		
* 1 st year*	101	29.4%
*2nd year*	50	14.6%
*3rd year*	115	33.5%
*4th year* +	54	15.7%
*Other*	23	6.7%

^*^NH Non-Hispanic

**Table 2:** Evaluation of implementation and impact of an interprofessional cannabis training, University of Arizona

	**Strongly agree**	**Agree**	**Neutral**	**Disagree**	**Strongly disagree**
	***n***(%)	***n***(%)	***n***(%)	***n***(%)	***n***(%)
This training addressed a training need for me in my current role	205 (59.4%)	106 (30.7%)	28 (8.1%)	4(1.2%)	2 (0.6%)
This training should be required for health care students in my profession	189 (54.8%)	96 (27.8%)	47(13.6%)	8(2.3%)	5(1.4%)
This training comprehensively addressed potential RISKS associated with cannabis use	266 (77.1%)	71 (20.6%)	5 (1.4%)	1 (0.3%)	2 (0.6%)
This training comprehensively addressed potential BENEFITS associated with cannabis use	225 (65.4%)	104 (30.2%)	11 (3.2%)	2 (0.6%)	2 (0.6%)
The training was inclusive of diverse experience and perspectives with regard to cannabis use	231(67.0%)	94(27.3%)	16(4.64%)	2(0.58%)	2(0.58%)
This training improved my knowledge of community resources that I can use	222 (64.5%)	101(29.4%)	17 (4.9%)	3 (0.9%)	1 (0.3%)
This training improved my ability to respond to patients interested in cannabis	238 (69.0%)	92(26.7%)	13 (3.8%)	1 (0.3%)	1 (0.3%)
As a result of this training: I am more likely to provide information to OTHERS to help them make informed choices about their use of cannabis	245 (71.2%)	84(24.4%)	11 (3.2%)	3 (0.9%)	1 (0.3%)
I learned information in this training that will help me in my work or community	241 (69.9%)	95 (27.5%)	6 (1.7%)	2 (0.6%)	1(0.3%)

**Table 3:** Selected trainee-reported learning outcomes of an interprofessional cannabis training, Comprehensive Center for Pain & Addiction, University of Arizona

	Trainee-reported learning outcomes*	**Degree program**
**Balanced, rigorous and non-ideological presentation of benefits, risks, and unknowns**	*“I learned how to balance information with common cannabis claims. For example, although overdose from cannabis may be rare, cannabis use impairs decision making and motor skills which lead to injury or death. I also learned the concept of supporting patient's self-directed decision making.”*	PharmD
	*“I learned that cannabis may have contaminants in them such as pesticides that can cause harm if not properly grown or regulated. It is important to know the source of where one gets the drug.”*	Nursing
	*[Synthetic forms of] cannabis are FDA-approved for chemotherapy induced n/v [nausea and vomiting] and increasing appetite in AIDS wasting.” [this comment references a discussion on Dronabinol and Epidiolex]*	Pharm D
	*“I learned that certain drugs interact with cannabis such as [Diazepam, lorazepam, and ondansetron].”*	Nursing
	*“Dispensary products are checked for accuracy of their labels, purity.”*	Pharm D
**Relevant and applicable to professional practice across disciplines**	*“I would like to share that I loved how the breakout rooms …allowed all health care professionals to understand each of our parts in caring for all patients. It really allowed me to grow my respect which is good for all healthcare workers.”*	Nursing
	*“I learned that different health care professionals view a scenario in different ways due to their training/experience.”*	Athletic Training
	*“This IPE also showed how important a collaborate team is when creating the best care plan for a patient. Our public health expert of the group offered a lot of insight for community outreach that I wouldn't have thought of without them!”*	PharmD
	*“There is a need for interprofessional relationships to be maintained so that these strategies are done smoothly and efficiently.”*	PharmD
**Person-centered practice intentions**	*“Across the life span, different health promotion strategies are necessary to communicate the risks of cannabis. An elder's personal knowledge from 40 years needs to be informed by more updated knowledge of potency.”*	Master’s Public Health
	*“I feel much better about informing my patients and even my family members about this alternative treatment, as well as the potential adverse effects and risks of using the medication. I hope soon, more research trials will continue to build our knowledge about Cannabis, its possibilities, and its risks.”*	Nursing
	*“Something that I took away from this insightful event was the stoplight method of helping patients determine where they are at in their level of cannabis use so that we, as nurses, can help them achieve their desired goals”*	Nursing
	*“Counsel them to not delay seeing their provider just because cannabis provides patients with temporary relief.”*	PharmD

^*^Bracketed phrases were added to contextualize trainee responses for clarity, as needed

#### Trainees agreed that the training comprehensively addressed potential risks and benefits

Nearly all (97.7%) of students either “agreed” or “strongly agreed” with the statement *“The training comprehensively addressed potential RISKS associated with cannabis use”*; 95.6% of students either “agreed” or “strongly agreed” with the statement *“The training comprehensively addressed potential BENEFITS associated with cannabis use”* (Fig. 1**, **Table 2). The Likert responses indicate that the training was experienced as comprehensive in terms of risks and benefits is supported by qualitative responses in which students cite increased knowledge of risks such as drug interactions, benefits of FDA-approved medication, as well as comments highlighting the importance of providing non-judgmental and evidence-based information to patients (Table 3).

#### Trainees agreed that person-centered communication approaches improved their capacity

94.2% of trainees “Agree” or “Strongly Agree” that *“The training was inclusive of diverse experience and perspectives with regard to cannabis use.”* Qualitative comments note the value of learning skills for stigma-free cannabis conversations with patients (Table 3). 93.9% of trainees “Agreed” or “Strongly agreed” that “*This training improved my knowledge of community resources that I can use*.” (Fig. 1, Table 2)*.*

#### Self-reported change in clinical capacity and intention to counsel

95.7% of trainees “Agreed” or “Strongly Agreed” that “*This training improved my ability to respond to patients interested in cannabis.*” (Fig. 1, Table 2). Nearly all (95.6%) students “Agreed” or “Strongly agreed” with the statement *“As a result of this training I am more likely to provide information to others to help them make their own informed decisions about cannabis.”*

Likert means from Year 1 and Year two were compared; no statistically significant differences were found.

## Discussion

The unique legal status of cannabis, including its contested and stigmatized history, the lack of rigorous research, and rapid expansion of consumer demand, presents challenges to the development and implementation of cannabis training in healthcare. An alarming gap in service quality is developing: health care professionals across disciplines lack the knowledge needed to educate and counsel patients, even as the prevalence of cannabis use rapidly accelerates. (Jankie et al., 2023, Ziemianski et al., 2015, Szaflarski et al., 2020) Few trainings exist, and even fewer are responsive to the nuances of a uniquely positioned substance that is rapidly being legalized for both therapeutic and recreational use. Yet, the small number of existing studies suggest that cannabis education has high potential to improve service quality. For example, Thant et al. found that exposure to cannabis education can improve providers’ confidence and knowledge to counsel patients. (Thant et al., n.d.) Clobes et al. found that educational interventions have the potential to reduce provider stigma. (Clobes et al., 2022).

Building on these foundational studies, we created this cannabis training as a first step in addressing critical gaps in healthcare provider knowledge related to cannabis. Project characteristics and trainee-reported measures suggest that the implementation strategies were effectively executed. Demand for the training was robust; each year, a second training date was added to accommodate student interest. Recruitment was especially strong among students in pharmacy, nursing, and public health. Representation among medical students was lagging. We identified that scheduling limitations faced by medical students dampened participation. To better meet their needs, the College of Medicine-Tucson is preparing to integrate the cannabis training content into the structured medical school curriculum. This training’s success has generated significant interest nationally and internationally.

We offer some possible considerations in support of replicability and adaptation of this model to other contexts. For our implementation goal of convening diverse expertise in curricular development, we acknowledge that our center’s focus on chronic pain and addiction positioned us to collaborate with experts in drug research, clinical practice, and patient-centered recovery. Institutions without immediate access to such collaborations might consider what incentives exist to form those partnerships institutionally, such as required teaching or service hours for faculty members. There is also the possibility of pursuing partnerships beyond their institution, including community-based resources or local governmental departments. Jurisdictions where cannabis has been legalized for medical or recreational use often fund cannabis education programs by non-profit organizations and as state or county health department initiatives and may be poised to contribute to developing such a training.

In interprofessional trainee recruitment, we also benefited from an existing interprofessional training infrastructure within our institution. Where such infrastructure is not in place, institutions may look to other opportunities to embed an interprofessional cannabis training within existing student symposia, seminars, or retreats, or wellness events. Grand rounds may also be a suitable venue, particularly if these can be opened to interprofessional attendance. Additionally, many advanced trainees may be interested in Continuing Education (CE/CME) credits for their respective licensures.

Lastly, when discussing potential risks and benefits and principles of person-centered care, we recommend a pragmatic approach acknowledging the reality of cannabis use among patients and the public at large. Grounding the training as a response to local policy contexts—such as qualified conditions for medical use, and recreational product trends—supports a neutral ideological stance that is focused on patient safety and outcomes. Additional ideological neutrality can be achieved by carefully contextualizing the interpretation of cannabis research in light of its status as a scheduled substance in the United States and many countries internationally.

### Limitations

Several limitations are noted. First, the possibility of self-selection bias cannot be completely ruled out, though our analysis comparing trainee evaluations from year 1 to year 2 did not find evidence in support of it. Second, the use of a generalizable rather than convenience sampling and/or the inclusion of a control group would improve the analytic rigor of this study; we are seeking funding toward this end. Third, while the training was demonstrably interprofessional and well-attended, medical students were underrepresented among participants due to logistical barriers inherent in medical education. Finally, further research will be needed to determine the extent to which the changes in behavioral intentions associated with the Cannabis IPE correspond with observable changes in professional practice, as well as to assess how long the knowledge and skills gained from the training may be retained. 

## Conclusion

We present our implementation and evaluation approach as a model to support the successful development and implementation of cannabis-related training for healthcare professionals. We would be remiss not to note here that the current funding (Torkamaneh 2024) of cannabis research is insufficient to address policy making and ensure consumer protection (Schauer 2024). Renewed commitment and investment will be required to ensure that rigorous, evidence-based research on cannabis can meet the rapidly expanding need, as well as evaluate educational interventions. This training is offered annually; we will continue to monitor and update the training to include current references and local, national, and international trends both in the presentation and in additional resources made available to the students. In future iterations, we hope to enrich our source material with data sources such as social media, Emergency Department admissions, updated clinical standards, and measurement of person-centered care as these become available.

## Supplementary Information

**Supplementary Material 1: Supplemental **Table 1**.** Selected references used during an interprofessional cannabis training, Comprehensive Center for Pain & Addiction, University of Arizona.

## Data Availability

This project was determined exempt by the University of Arizona Human Subjects Protection Program, 04/24/2023, STUDY00002810. De-identified data are available upon reasonable request to the corresponding author.
