# Supplementary material for: “It’s legal, now what?” development, implementation, and evaluation of interdisciplinary cannabis education for healthcare trainees
Source: J Cannabis Res. 2025 Sep 26;7:68. doi: 10.1186/s42238-025-00321-8 (PMC12465847; doi:10.1186/s42238-025-00321-8)
Supplement: Supplementary file 1 — Supplementary Material 1: Supplemental Table 1. Selected references used during an interprofessional cannabis training, Comprehensive Center for Pain & Addiction, University of Arizona. [file 42238_2025_321_MOESM1_ESM.docx]

**Supplemental Table 1:** Selected references used during an interprofessional cannabis training, University of Arizona Health Sciences

| **Facts on Cannabis consumption in the US** |
| --- |
| Centers for Disease Control and Prevention. Facts on Cannabis consumption in the US [Internet]. Atlanta (GA): CDC; [cited 2025 May 22]. Available from: <https://www.cdc.gov/cannabis/data-research/facts-stats/index.html> |
| **Facts on Cannabis consumption, Markets in the United States (includes age groups and use)** |
| National Academies of Sciences, Engineering, and Medicine; Health and Medicine Division; Board on Population Health and Public Health Practice; Committee on the Public Health Consequences of Changes in the Cannabis Policy Landscape; Boyle EB, Hurd YL, Teutsch SM, editors. *Cannabis Policy Impacts Public Health and Health Equity. 3, Cannabis Consumption and Markets in the United States*. Washington (DC): National Academies Press (US); 2024. Available from: <https://www.ncbi.nlm.nih.gov/sites/books/NBK609482/> |
| Baldwin GT, Vivolo-Kantor A, Hoots B, Roehler DR, Ko JY. Current Cannabis Use in the United States: Implications for Public Health Research. *Am J Public Health*. 2024;114:S624–S627. doi:10.2105/AJPH.2024.307823 |
| **Cannabis and Work: Implications, Impairment, and the Need for Further Research** |
| National Institute for Occupational Safety and Health. Cannabis and Work: Implications, Impairment, and the Need for Further Research [Internet]. CDC Blogs; 2020 Jun 15 [cited 2025 May 22]. Available from: <https://blogs.cdc.gov/niosh-science-blog/2020/06/15/cannabis-and-work/> |
| Crean RD, Crane NA, Mason BJ. An evidence based review of acute and long-term effects of cannabis use on executive cognitive functions. *J Addict Med*. 2011;5(1):1–8. doi:10.1097/ADM.0b013e31820c23fa. PMID: 21321675; PMCID: PMC3037578. |
| **Driving and cannabis use** |
| Sewell RA, Poling J, Sofuoglu M. The effect of cannabis compared with alcohol on driving. *Am J Addict*. 2009;18(3):185–93. doi:10.1080/10550490902786934. PMID: 19340636; PMCID: PMC2722956. |
| Pearlson GD, Stevens MC, D'Souza DC. Cannabis and Driving. *Front Psychiatry*. 2021;12:689444. doi:10.3389/fpsyt.2021.689444. PMID: 34630173; PMCID: PMC8499672. |
| National Institute of Justice. Field Sobriety Tests and THC Levels Unreliable Indicators of Marijuana Intoxication [Internet]. Washington (DC): NIJ; [cited 2025 May 22]. Available from: <https://nij.ojp.gov/topics/articles/field-sobriety-tests-and-thc-levels-unreliable-indicators-marijuana-intoxication> |
| **Blood levels of THC in humans** |
| Wurz GT, DeGregorio MW. Indeterminacy of cannabis impairment and ∆9-tetrahydrocannabinol (∆9-THC) levels in blood and breath. *Sci Rep*. 2022;12:8323. doi:10.1038/s41598-022-11481-5 |
| **Mechanisms of action and Drug-Drug interactions of Cannabis products.** |
| Sharma P, Murthy P, Bharath MM. Chemistry, metabolism, and toxicology of cannabis: clinical implications. *Iran J Psychiatry*. 2012;7(4):149–56. PMID: 23408483; PMCID: PMC3570572. |
| Chayasirisobhon S. Mechanisms of Action and Pharmacokinetics of Cannabis. *Perm J*. 2020;25:1–3. doi:10.7812/TPP/19.200. PMID: 33635755; PMCID: PMC8803256. |
| Smith RT, Gruber SA. The complex relationship between cannabinoids and hepatic metabolism resulting in the potential for drug-drug interactions. *Front Psychiatry*. 2023;13. doi:10.3389/fpsyt.2022.1055481 |
| Farokhnia M, McDiarmid GR, Newmeyer MN, et al. Effects of oral, smoked, and vaporized cannabis on endocrine pathways related to appetite and metabolism: a randomized, double-blind, placebo-controlled, human laboratory study. *Transl Psychiatry*. 2020;10:71. doi:10.1038/s41398-020-0756-3 |
| Nasrin S, Watson CJW, Perez-Paramo YX, Lazarus P. Cannabinoid Metabolites as Inhibitors of Major Hepatic CYP450 Enzymes, with Implications for Cannabis-Drug Interactions. *Drug Metab Dispos*. 2021;49(12):1070–80. |
| Bardhi K, Coates S, Watson CJW, Lazarus P. Cannabinoids and drug metabolizing enzymes: potential for drug-drug interactions and implications for drug safety and efficacy. *Expert Rev Clin Pharmacol*. 2022;15(12):1443–60. doi:10.1080/17512433.2022.2148655 |
| **Cannabis contaminants: sources, distribution, human toxicity and pharmacologic effects.** |
| Dryburgh LM, Bolan NS, Grof CPL, Galettis P, Schneider J, Lucas CJ, Martin JH. Cannabis contaminants: sources, distribution, human toxicity and pharmacologic effects. *Br J Clin Pharmacol*. 2018;84(11):2468–76. doi:10.1111/bcp.13695. PMID: 29953631; PMCID: PMC6177718. |
| Montoya Z, Conroy M, Vanden Heuvel BD, Pauli CS, Park S-H. Cannabis Contaminants Limit Pharmacological Use of Cannabidiol. *Front Pharmacol*. 2020;11. doi:10.3389/fphar.2020.571832 |
| Jameson LE, Conrow KD, Pinkhasova DV, Boulanger HL, Ha H, Jourabchian N, et al. Comparison of State-Level Regulations for Cannabis Contaminants and Implications for Public Health. *Environ Health Perspect*. 2022;130(9):97001. doi:10.1289/EHP11206. PMID: 36102653; PMCID: PMC9472674. |
